# Lessons Learned from In-Person Conferences in the Times of COVID-19

**DOI:** 10.3390/ijerph20010510

**Published:** 2022-12-28

**Authors:** Maryam Ehteshami, Carlos León Edgar, Lucia Yunuen Delgado Ayala, Michael Hagan, Greg S. Martin, Wilbur Lam, Raymond F. Schinazi

**Affiliations:** 1 Center for ViroScience and Cure, Laboratory of Biochemical Pharmacology, Department of Pediatrics, Emory University, 1760 Haygood Drive, Room E418, Atlanta, GA 30322, USA; 2 WeCare Clinic, Carr. Transpeninsular Km 24.5 Consultorios H+ Koral Center, Cerro Colorado, San José del Cabo 23406, Mexico; 3 Informed Horizons Education, 1860 Montreal Road, Tucker, GA 30084, USA; 4 School of Medicine, Emory University, Atlanta, GA 30322, USA; 5 Children’s Healthcare of Atlanta, Atlanta, GA 30322, USA; 6 Georgia Institute of Technology, Atlanta, GA 30332, USA

**Keywords:** COVID-19, scientific meeting, in-person conferences, rapid antigen testing, risk mitigation, Hep-DART

## Abstract

Scientific societies and conference secretariats have recently resumed in-person meetings after a long pause owing to the COVID-19 pandemic. Some safety measures continue to be implemented at these in-person events to limit the spread of severe acute respiratory syndrome coronavirus 2 (SARS-CoV-2). With increased numbers of waves of infection, caused by the emergence of SARS-CoV-2 variants, additional information is needed to ensure maximal safety at in-person events. The MEX-DART case study was conducted at the in-person Hep-DART 2021 conference, which was held in Los Cabos, Mexico, in December 2021. Many COVID-19 safety measures were implemented, and incidence of SARS-CoV-2 infection during the conference was tested onsite. In this study, we highlight the specific conditions and safety measures set in place at the conference. In addition to vaccination requirements, social distancing, and mask wearing, daily rapid testing was implemented for the duration of the conference. At the end of the 4-day meeting, none of the 166 delegates (and family members attending the conference) had tested antigen positive for SARS-CoV-2. Two delegates tested positive in the week after the conference; the timing of their positive test result suggests that they contracted the virus during their travels home or during postconference vacationing. We believe that this model can serve as a helpful template for organizing future in-person meetings in the era of COVID-19 and any other respiratory virus pandemics of the future. While the outcomes of this case study are encouraging, seasonal surges in respiratory virus infections such as SARS-CoV-2, RSV, and influenza virus incidence suggest that continued caution is warranted.

## 1. Introduction

With the onset of the COVID-19 pandemic in early 2020, many in-person events, including scientific meetings and workshops, were canceled, postponed, or delivered virtually. As vaccination becomes more widespread and severe acute respiratory syndrome coronavirus 2 (SARS-CoV-2) incidence is reduced, more face-to-face events are starting to take place. It is therefore important to highlight newly adopted COVID-19 safety measures employed at in-person medical conferences and examine their impact on transmission and outbreak outcomes. Since the start of the pandemic, several studies have examined the impact of social distancing, ventilation, and other safety requirements on reducing transmission and preventing outbreaks at closed-space events such as conferences [1,2], concerts [3,4], dancing clubs [5], and other social events [6,7,8]. In this study, we highlight specific mitigation measures applied at the Hep-DART 2021 meeting, with the goal of highlighting best practices, which may impact future in-person meetings.

Hep-DART is a biannual meeting designed to provide a state-of-the-art forum for cutting-edge research on hepatology, viral hepatitis, and chronic liver disease. With a special emphasis on discovery and the development of life-saving therapeutics, this meeting promotes interaction and collaboration between basic science researchers, clinicians, and community partners with the goal of advancing knowledge on liver disease treatments, management, and cures. Hep-DART counts among its faculty Nobel prize winners who codiscovered the hepatitis C virus, and world-renowned scientists who subsequently discovered the cure for this devastating infection. Always at the forefront of scientific discovery, in recent years, Hep-DART has shifted its focus to hepatology and chronic hepatitis B, which affects over 300 million individuals, and chronic fatty liver disease, which is a subject of much recent discovery and development. Given the fast pace of this scientific field, Hep-DART meetings are recognized for providing a state-of-the-art platform for scientific exchange. It was a great concern when all in-person meetings were postponed because of the COVID-19 pandemic.

Hep-DART 2021 was originally planned to take place in early December 2021 at Hilton Los Cabos, in Mexico. Owing to the ongoing pandemic, both virtual and in-person meeting options were simultaneously developed in order to accommodate the meeting in the safest manner possible. As the date of the conference approached, SARS-CoV-2 incidence was decreasing, and vaccination rates were increasing; therefore, it was deemed possible to move forward with an in-person meeting, albeit under the strictest safety measures. In this case study, we highlight the specific conditions and mitigation measures set in place that led to the successful delivery of Hep-DART 2021. This case study will detail all the measures taken to maximize health and safety. These include logistical considerations, social distancing, masking, and vaccination policies, as well as the implementation of onsite testing. We believe that our model serves as a helpful template for organizing future in-person meetings in the era of COVID-19.

## 2. Materials & Methods

Conference demographics. The Hep-DART 2021 meeting took place during 5–9 December 2021 at the Hilton Los Cabo in San Lucas, Mexico. Informed Horizons Education Inc. (IHE, Tucker, Georgia, USA), a nonprofit medical education company, served as conference secretariat and managed the logistical aspects of the meeting. The organizing committee worked closely with IHE and Hilton Los Cabos management to implement a series of complementary safety measures to reduce the risk of COVID-19 infection (summarized in Table 1).

**Figure 1:**
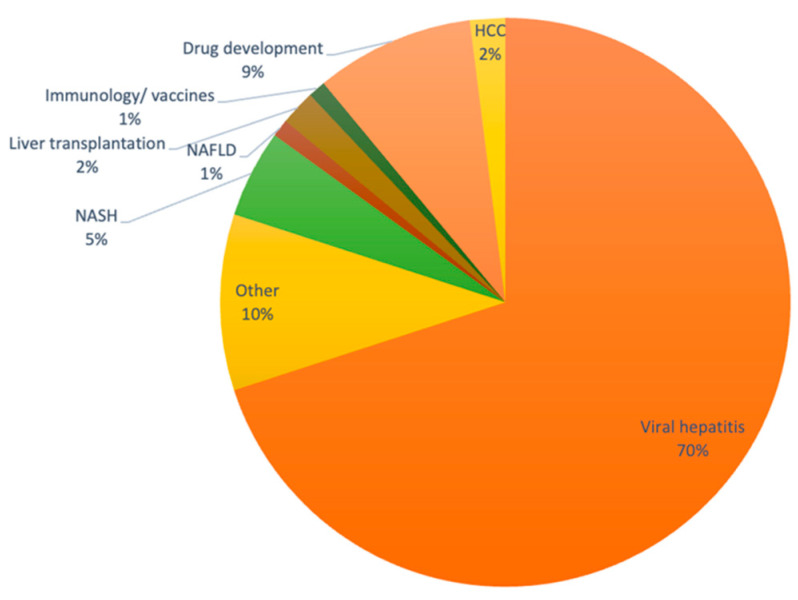
Hep-DART demographics. Area of expertise for conference attendees (N =148). Abbreviations: HCC: Hepatocellular carcinoma; NAFLD: Non-alcoholic fatty liver diseases; NASH: Non-alcoholic steatohepatitis.

Hep-DART 2021 brought together global stakeholders to share research results, important clinical developments, and updates on ongoing and new trials in the field of hepatology. The program included 46 invited lectures, 12 oral abstract presentations, 4 roundtable discussions, and 1 debate session. In total, 25.5 h were spent in session indoors over the course of this 5-day meeting. The program attracted 166 delegates from 20 countries (Table 2), plus 10 accompanying guest registrations. Most of the participants were academic researchers (27.3%) or physicians (27.3%), and the remaining 45.4% were either corporate professional, nurses, consultants, pharmacists, students, or “others”. As summarized in Figure 1, 70% of the respondents self-identified as experts on viral hepatitis, drug development (9%), or other specialties (21%). Figure 1 highlights the area of expertise of attending delegates.

Safety measures prior to the start of the conference: COVID-19 stats and conditions leading up to the pandemic. We carefully monitored trends in SARS-CoV-2 incidence in the months leading up to the conference. Knowing that most Hep-DART 2021 delegates were coming from the US, we closely monitored trends in new infections across the US. Given that the trends were steady, we decided to move forward with the in-person meeting. At the time, Mexico did not require any COVID-19 testing for incoming travelers. However, we instated a policy that all conference delegates attest to having received their vaccination and that they would perform a rapid test onsite upon arrival (please see next section for additional details). The first cases of the Omicron variant were being identified in the US just days before the start of the conference. By 3 December, the number of infections were increasing, but no new changes to travel guidelines were announced in the US. Canada introduced new travel restrictions on 1 December 2021, which led to one invited speaker’s cancelation [9]. This invited speaker was traveling from Egypt, with a layover stay in Canada, and she had to cancel her attendance. The lecture was instead delivered by a collaborator who was present at the meeting. Additionally, thanks to the use of digital conference programming and an onsite mobile app, the changes in the scientistic program were readily communicated to the delegates. Local print shops were also solicited to print paper copies of the updated agenda to be made available onsite. Ultimately, Omicron peaked in the US on 15 January 2022, well after the end of Hep-DART 2021 [10]. Had Hep-DART 2021 been held a few weeks later, we would have reverted to the virtual option.

Onsite safety measures during the conference. To maximize safety, we adopted a multipronged approach. Current literature indicates that the combination of several safety measures, often described as the “Swiss cheese model” [11,12,13], provides the maximal level of protection. Briefly, the Swiss cheese model conceptualizes that transmission can be reduced when layered mitigation strategies, each intervention targeting unique modes of transmission, are implemented. As described below, we adopted several of these measures, including vaccination, testing, and social distancing (Table 1).

Vaccination policy. We asked that all attendees bring proof of vaccination. If a delegate was not vaccinated, for the safety of other delegates, they would have been subjected to daily antigen testing. When registering for the meeting online, delegates were asked to fill out a survey indicating whether they had been vaccinated. Through the questionnaire, the delegates had the option to choose one of the following responses: (1) not vaccinated; (2) fully vaccinated (two weeks after the final dose); or (3) partially vaccinated. Of the 166 delegates registered, all attested that they were fully vaccinated.

Testing. Presenting a negative COVID-19 test result was not mandatory to enter Mexico. However, all passengers, vaccinated and nonvaccinated, may have been subject to health screenings, including temperature checks upon arrival, and those exhibiting symptoms would have been subject to additional health screening and/or quarantine. At the time of the conference, self-administered rapid tests were not ubiquitously available. Therefore, we partnered with a local clinic to provide nurse-administered rapid antigen testing for all delegates and their travel companions onsite. All delegates had to show proof of a negative test result prior to entering conference halls on the first day of the meeting. Free testing was also offered on a voluntary basis throughout the 5-day conference. To provide rapid testing to all delegates, we partnered with Abbott Laboratories and local healthcare providers. Briefly, 1500 free rapid tests were donated by Abbott Laboratories (Panbio COVID-19 Ag Rapid Test Device; Lot number 41ADG666A). We contracted a professional local clinic to administer the test for the duration of the conference. A testing site was set up adjacent to the hotel lobby, and a medical doctor and a nurse were available for a minimum of 3 hours a day to administer the test to conference delegates and their travel companions. Test results were sent to each delegate via email or printed on paper immediately after testing. All delegates had to show proof of a negative test result before entering the conference hall on the first day of arrival. In order to speed up the verification process, all delegates showed their negative test result at the registration desk, at which point they received a colored sticker on their name badge. In this way, all delegates were made aware of each delegate’s test result status. Testing was also available on all 5 days of the conference. Once the delegates had completed their day 1 test, all subsequent tests were voluntary. Testing was also provided 1 day after the conference for those who needed it for their return home. Testing was also made available to hotel staff at no extra charge. In cases where a PCR test was needed, the onsite nurse was able to collect the samples and send them to a local laboratory for testing. PCR test results were available withing several hours, no later than 24 h. Results of the rapid testing were collected and anonymized for analysis in this study.

Venue, social distancing, and mask wearing. Historically, the meeting capacity has been between 300 and 400 delegates. This year, in order to be able to set up the venue space with maximal social distancing, we capped registration at 200 delegates. Conference sessions were held in the El Dorado Ballroom (total size 7445 square ft (692 sq meters) with a ceiling height of 21.48 feet (6.5 m)). Under normal conditions, the ballroom allows for 510 persons seated in a “classroom style” (chairs and tables). Because of the requirements of social distancing, we reduced the number of seats available to 200. This allowed for increased spacing between each seat. Instead of the usual three chairs per 8 ft (2.4 m) table, only two chairs were placed at each table. This allowed for a distancing of ~2 ft, 8.5 inches (0.74 m) between each chair, in all directions. Taking into account the square footage of the ballroom 7445 sq ft (692 sq meters), divided by the maximal number of delegates (166), the density of the room was calculated to be 44.8 sq ft/person (4.2 sq meter/person). Further, mask wearing was strictly required at all indoor events. Complementary KN95 masks were made available at the registration desk, positioned outside of the main conference hall, and conference staff actively monitored mask wearing throughout the meeting sessions. All surfaces, including the podium and microphones, were disinfected between each talk, and disinfectant wipes and hand sanitizers were made available throughout the conference venue.

All meals, including breakfasts, lunches, receptions, and all AM and PM breaks, were planned in an outdoor setting. Unlike previous years, the poster reception was also set up outdoors, with a reduced number of posters per board to minimize crowding. For outdoor events, mask wearing was strongly encouraged, especially when not eating.

Quarantine measures. We worked closely with the hotel to implement a quarantine protocol in case of a positive COVID-19 test. The protocol included asking the delegate to stay in their room until two negative antigen tests had been obtained. The hotel agreed to offer a reduced room rate for the duration of the quarantine. Delegates would receive a special discount on food and beverage as well and were not permitted to leave their rooms while testing positive unless recommended by a doctor. Room service and housekeeping packages were arranged to be left outside of the guest room.

Data collection. In order to collect and analyze the test results, we partnered with WeCare, a Mexican clinic who performed the test swabs. The medical staff did not require an IRB for this study, but they drafted the informed consent forms that were needed to ensure proper consent for data collected was received from each participant. 

## 3. Results

**Figure 2:**
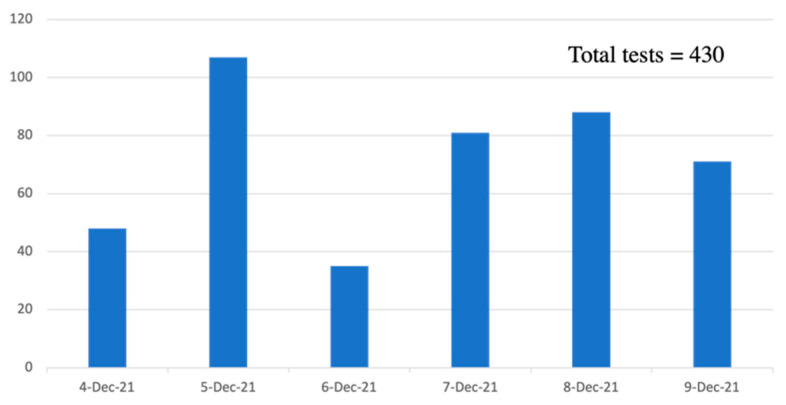
Number COVID-19 rapid antigen tests performed at conference venue. December 4, 2021 (48 tests), December 5, 2021 (107 tests), December 6, 2021 (35 tests), 7 December 2021 (81 tests), 8 December 2021 (88 tests), 9 December 2021 (71 tests). Total tests performed = 430 rapid tests.

Prior to taking their COVID-19 rapid test, conference delegates were informed of the MEX-DART study and were given the option to participate after reviewing and signing the consent form. The consent form was made available in English and Spanish, both on paper and digitally (via QR code scanning). Delegates also had the option to have their rapid test conducted onsite without participating in the study. The goal of the MEX-DART study was to limit or prevent the spread of the virus at this scientific conference. However, because not a single delegate tested positive, the study did not provide powered quantitative data. Nonetheless, the results of the onsite testing are provided herein. Over the 6 days when onsite testing was made available, 430 rapid tests were performed. Figure 2 highlights the number of tests performed per day at the conference venue.

Of the 430 tests performed, only one individual’s rapid test showed a positive result, on 8 December 2021. Upon identification of this test result, the individual was immediately sent to their hotel room for isolation. A second PCR test was performed from the hotel room. While conference staff awaited the PCR results, other conference delegates who were identified to have had close contact with the individual were rapidly tested and isolated in their hotel rooms. The hotel staff servicing the potentially infected individual’s room were also identified, isolated, and tested. Within 3 h after the initial positive rapid test, the PCR results came back negative. Repeat testing confirmed that the initial positive rapid test was indeed a false positive. No other individuals tested positive for COVID-19 for the duration of the conference.

Upon conference conclusion, delegates were given a take-home rapid test kit and were invited to notify the meeting organizers in case of a positive result. A postconferenece survey was electronically disseminated to confernece registrants in order to capture any positive cases identified through self-reporting. Of the 52 survey respondants, two individuals subsequently self-identified as having tested positive in the week after the meeting. In one case, the delegate reported testing positive on Sunday, 12 December 2021. This individual described that they stayed in Cabo San Lucas for an extra day and attended several indoor restaurants and bars in the city center. In line with the timing of the positive test, we believe that the individual contracted the virus during their excursions into the city. The second individual reported that they tested positive on Tuesday, 14 December, and indicated that their exposure may have happened during air travel home. No other individuals self-identified as having tested positive in the week after the conference.

## 4. Discussion

Overall, the lack of detection of any positive SARS-CoV-2 cases during the conference provides strong evidence that mitigation measures implanted during Hep-DART 2021 were effective in curbing SARS-CoV-2 infection among the delegates. Given that the first wave of Omicron was just taking off internationally, we believe that the safety measures upheld at the meeting helped keep the virus at bay. It is difficult to determine which safety measure delivered the most benefit. In addition to high vaccination rates being a critical component of health safety [14], we believe that the Swiss cheese model [11,12,13] for risk mitigation adopted at Hep-DART 2021 significantly impacted the success of this meeting. Of note, we were able to adjust the social and scientific program of the meeting to maximize time spent outdoors. This is especially true for the poster reception, which has historically been held indoors and usually generates dense crowds around each poster board. Although outdoor space was available for the posterboard at Hep-DART 2021, special weighted sacs had to be brought in to protect the boards from strong winds. Rain was not a concern, because December in Cabo is usually dry. The size of this conference allowed for ample social distancing, which has been shown to be an effective mitigation measure [15,16,17]. For larger conferences, space limitations need to be considered to make sure that the chosen venue has the capacity to accommodate social distancing, relative to the number of registrants. Finally, in December 2021, regular travel had not yet reached pre-COVID-19 levels. Therefore, the number of nondelegate guests were at a minimum at the conference hotel. Future conferences should take into account that as travel levels return to normal, social distancing in public areas of the hotel venue may prove more challenging. In terms of delegate compliance, we observed that almost all participants readily complied with the COVID-19 conference rules and in fact expressed satisfaction with the measures taken to protect their health and those of their companions and hotel staff. Mask wearing compliance was reinforced at all times. This measure has been shown to be effective in mitigating SARS-CoV-2 transmission [18,19]. Additionally, 90% of delegates self-described as conducting research, or practicing medicine, in areas related to infectious diseases (Figure 1). Therefore, it is possible that the Hep-DART 2021 delegation was particularly compliant with COVID-19 measures. It remains to be seen whether conferences where most delegates do not have a strong knowledge of infectious diseases and virology would be as compliant with safety measures. In terms of onsite testing, overall, both delegates and meeting organizers were satisfied with the practicality of having tests conducted onsite. One limitation was the long queue formation on the first day of the conference as all incoming participants needed to get tested before entering the conference venue. To our knowledge, no waiting time exceeded 20 min, and most delegates were able to attend the opening session without delay, though there were a handful of instances where the electronic test result delivered via email was delayed. In the future, concerns related to delays in receiving test results may be addressed by providing the delegates with self-tests, which would not require a visit to the testing station.

A possible limitation of the testing setup described here relates to the sensitivity of rapid antigen tests. It has been reported that serial rapid testing will improve the accuracy of testing [20]. All conference delegates were required to complete at least one test upon entry, but subsequent tests were left at the discretion of each participant. This opened up the possibility to a positive case’s going undetected for longer than desired. In the future, we are considering making daily self-testing an option, depending on the availability of rapid tests and possibly influenced by community incidence of SARS-CoV-2 at the time of the event. Another limitation of the study relates to the self-reported nature of the postmeeting survey. Only 52 out of 166 registrants responded to this survey. The onsite testing results that we accumulated allowed us to fill in the gaps and get a more in-depth picture of SARS-CoV-2 status of the attendees for the duration of the conference.

## 5. Conclusions

In conclusion, the countermeasures applied at Hep-DART 2021 were beneficial in maximizing safety for all participants. Although one measure is likely insufficient to prevent SARS-CoV-2 infection, the combination of measures, such as social distancing, outdoor planning, vaccination, masking, and regular testing, maximize opportunities for returning to in-person conference in a safe, yet scientifically stimulating environment. This study strongly supports the benefits of adopting a multipronged approach to COVID-19 safety measures and risk mitigation. Given that the pandemic is still ongoing and that the emergence of new variants is likely, preventing outbreaks at future in-person scientific meetings is essential. The lessons learned from this experience can be applied to future respiratory virus epidemics or pandemics, including those from SARS-CoV-2, RSV, and the influenza virus.

## Figures and Tables

**Table 1:** Summary of COVID-19 safety measures at Hep-DART 2021.

Mitigation Measure	Rationale and Details
Vaccination	All attendees provided attestation of vaccination prior to arrival at the conference venue. 100% of participants attested to being vaccinated
Onsite testing	Rapid Antigen tests were donated by Abbott Laboratories. Local clinic set up a testing site in the hotel lobby. All delegates were tested upon arrival and had to show a proof of negative test before entering conference halls. Testing was also made available for the entire duration of the conference, as well as a day post-meeting.
Limited capacity and social distancing	Reduced capacity allowed for increased social distancing. The 510-person conference hall was set up with 200 chairs (classroom style) to allow for maximal distancing.
Mask wearing	Masks have shown to be an effective mitigation measure against the spread of SARS-CoV-2. Mask wearing was strictly enforced throughout all indoor events. For outdoor events, delegates were required to wear a mask at all times except while eating.
Maximizing outdoor events	All meals were served outdoors. The poster reception was also held outdoors and at reduced capacity. Delegates were asked to wear masks at outdoor events except while eating.

**Table 2:** Hep-DART 2021 delegate demographics by country.

Region	Participants	Distribution
Australia	1	0.65%
Austria	2	1.29%
Azerbaijan	1	0.65%
Belgium	2	1.29%
Brazil	1	0.65%
Bulgaria	1	0.65%
Canada	9	5.81%
Egypt	1	0.65%
France	3	1.94%
Germany	2	1.29%
Hong Kong	1	0.65%
Italy	2	1.29%
Mexico	5	3.23%
Russian Federation	1	0.65%
Singapore	1	0.65%
South Korea	1	0.65%
Sweden	3	1.94%
United Kingdom	2	1.29%
United States	116	74.84%

## Data Availability

The data used for this analysis can be made available upon reasonable request to corresponding author.
